# Effectiveness of a Patient Support Program in Supporting Access to Therapy for Solid Tumor Malignancies

**DOI:** 10.36469/9846

**Published:** 2016-10-27

**Authors:** Lindsay Parker, Alissa Shaul, Brian Murphy, Zeba M. Khan

**Affiliations:** 1 Evidera, Lexington, MA; 2 Celgene Corporation, Summit, NJ

**Keywords:** free medication program, appeals support, prior authorization, co-payment assistance, solid tumor malignancies, patient support program

## Abstract

**Background:** Certain governmental agencies, patient advocacy organizations, and pharmaceutical manufacturers have implemented programs to assist patients in overcoming barriers to accessing healthcare. Recently, such programs have expanded their services, helping both uninsured and insured patients to navigate the complex healthcare system, and assisting with increasing out-of pocket costs and copays for the drugs.

**Objective:** To better understand the effect of patient support programs on access to therapy for solid tumor malignancies, this study evaluated service use, case outcomes, and patient characteristics from a manufacturer-sponsored program in the United States.

**Methods:** Sociodemographic characteristics, services use rates, and outcomes by case and insurance type were evaluated at the patient- and case-level in a random sample of patients prescribed nab-paclitaxel for solid tumor malignancies who enrolled in the Celgene Patient Support (CPS) program (April 2011–November 2013).

**Results:** This analysis included 4566 patients (8134 cases); most patients were female (64.7%), aged <65 years (59.2%), in the South (53.9%), and treated in community settings (87.9%). Patients were primarily insured by Medicare (38.5%) or commercial plans (37.3%), or were uninsured (16.6%). Following benefits investigations for new patients entering the program (98.5%), CPS provided support to obtain free medicine (29.4%), appeal denial of coverage (15.0%), receive commercial co-payment assistance (8.1%), or obtain prior payer authorization (1.3%). Nab-paclitaxel was provided at no cost in 89.4% of cases where patients sought financial support; payer reimbursement was obtained in 63.2% of reimbursement appeals. Of commercially-insured patients who required assistance with co-payments and met financial criteria, 93.3% received a mean of $597 in co-payment support.

**Conclusion:** The CPS program was successful in gaining access to therapy. Healthcare providers and both insured or uninsured patients accessed CPS for prior authorization/precertification, appeals support, and financial support through the Free Medication Program and the Commercial Co-Pay program.

## INTRODUCTION

Access to effective treatment is a challenge for patients with cancer in the United States (US) due to the heterogeneity and limitations of the current healthcare insurance system.[1] Some patients are uninsured or have insurance benefits that do not adequately cover cancer treatments.[2,3] In addition, the separation of medical and pharmacy benefits and dissimilar cost-sharing mechanisms affect patients differently depending on whether they are seeking intravenous or oral treatment.[3] Thus, the complex and varied policies of insurance plans pose barriers to patients attempting to deal with their clinical condition and the challenges of their particular insurance situation.[1,3]

Affordability and access to treatment are major issues for cancer patients. Financial constraints may be heightened as co-payments accumulate over the course of disease, perhaps associated with regular treatment cycles for extended periods.[4] Many cancer patients experience financial and psychological strain associated with managing high out-of-pocket costs, insurance-related administrative tasks, and fear of losing coverage or delayed or denied authorization.[1,5-7]

These affordability and access issues can have serious implications on disease outcomes and health-related quality of life. Barriers to therapy may cause treatment delays or interruptions, and contribute to sub-optimal medication adherence.[8] More than one million patients with cancer are suspected of foregoing care due to high co-payments.[4] Although the implementation of the Patient Accountability and Affordable Care Act may improve access to care, it may not necessarily reduce the economic barriers to quality cancer care.[9]

In response to these needs, certain governmental agencies, patient advocacy organizations, and pharmaceutical manufacturers have implemented programs to assist patients in overcoming barriers to accessing healthcare. Traditionally, patient assistance programs focused largely on uninsured patients needing financial support for cancer drugs.[4,8,10-14] Recently, such programs have expanded their services, helping both uninsured and insured patients to navigate the complex healthcare system, and assisting with increasing out-of pocket costs and copays for the drugs.[1,3,4,15] However, lack of patient awareness of how to apply for and successfully use such services presents a hurdle to the effectiveness of support programs.

An example of a support program with comprehensive services is the Celgene Patient Support® (CPS) program created in 2007 by Celgene Corporation.[3,16] This national program provides patients and healthcare professionals with a central point of contact to assist with identifying financial assistance options and obtaining insurance approval for patients who use Celgene products.[3] A dedicated CPS® Specialist is assigned to each incoming assistance request, ensuring personal access and reimbursement support. The CPS team of Celgene employees includes nurses, pharmacy technicians, and social workers—all dedicated to the patients they serve. They investigate and explain health insurance benefits, navigate Medicare and other coverages, facilitate prior authorizations, assist with appeal support after insurance denials, assess copayment options, monitor pending prescriptions status, administer the process of applying for free medication, and guide patients through restricted-distribution programs. The goal of the CPS is to match individual patients with applicable coverage opportunities as quickly as possible to ensure that patients who are prescribed medicines receive them.[3] Four out of five patients who requested help from CPS received their Celgene medication.[17]

Since 2007, CPS has helped more than 50,000 patients and provided more than $750 million in free medication. Most patients are referred to the CPS program by healthcare professionals, and healthcare professionals and patients voluntarily choose to participate in the program.[3] Some patients contact the program directly as a result of information received from oncology societies, advocacy groups, foundations, local support groups, or via their own internet-based research. The CPS does not conduct any direct-to-consumer advertising.[3]

To better understand the impact of patient support programs on treatment access, the current study evaluated the effect of the CPS program on patients' access to nab-paclitaxel (Abraxane®, Celgene Corporation, Summit, NJ 07901). It is a microtubule inhibitor with three indications: 1) treatment of metastatic breast cancer, after failure of combination chemotherapy for metastatic disease or relapse within six months of adjuvant chemotherapy; 2) first-line treatment of locally advanced or metastatic non-small cell lung cancer with carboplatin, in patients who are not candidates for curative surgery or radiation therapy; and 3) first-line treatment of patients with metastatic adenocarcinoma of the pancreas, in combination with gemcitabine.[18] This study sought to determine the characteristics of the patients who enroll in the CPS program, the service components that those patients request and receive, and how those components contribute to the effectiveness of the program.

### METHODS

#### Data Sources and Study Population

De-identified patient information was obtained through the CPS database, in which trained Specialists recorded the type of assistance sought by and provided to patients with solid tumors prescribed nab-paclitaxel. The study cohort represented a random sample of patients enrolled in the CPS program from April 1, 2011 to November 1, 2013.

#### Measures and Analysis

The analysis was performed using patient-level data from the CPS database for sociodemographic characteristics (e.g., age, gender, geographic region, and type of provider, case, primary insurance plan); insurance type (e.g., Medicare, Medicaid, commercial, military, state program, no insurance, other commercial, other); and case type and outcomes of assistance sought by patients (benefits investigation, prior authorization/precertification, appeals support, Free Medication Program, or Commercial Co-pay Program). Individual patients may have one or more cases within the CPS database. Most patients' initial assistance requests to CPS led to benefits investigations which, depending on those outcomes, may have led to the initiation of another case type (i.e., prior authorization, appeals support, Free Medication Program, or Commercial Co-pay Program).

The data were summarized by descriptive statistics at the patient level for demographics and at the case level for outcomes of assistance requests. Statistics were obtained for patients or cases overall, and by insurance plan type and case type. Categorical variables were tabulated as the number and proportion of patients or cases, and continuous variables as mean and median values with standard deviations (SD). The analyses were conducted using SAS statistical software version 9.2 (SAS Institute Inc., Cary, NC).

### RESULTS

#### Sample Patient Characteristics

This analysis included a random sample of 4566 patients prescribed nab-paclitaxel for solid tumor malignancies who enrolled in the CPS program during April 2011 to November 2013. A total of 8134 cases were reported for these patients. Table 1 summarizes the sociodemographic characteristics of CPS program patients overall and by case type.

The mean (±SD) patient age was 61.7 (11.9) years. For all case types combined, a majority of the patients were aged <65 years (59.2%) and female (64.7%). Approximately 53% of patients lived in the South census region (Alabama, Arkansas, Delaware, District of Columbia, Florida, Georgia, Kentucky, Louisiana, Maryland, Mississippi, North Carolina, Oklahoma, South Carolina, Tennessee, Texas, Virginia, and West Virginia). Also, 87.9% of patients received their cancer care in a community clinical setting (versus an academic or university setting). The patients varied widely with respect to their insurance coverage, with the plurality insured by Medicare (38.5%; n=1,756), followed by commercial insurance (37.3%; n=1702), no insurance (16.6%; n=755), and Medicaid (8.7%; n=397).

To determine whether characteristics of patients prescribed nab-paclitaxel differed by the type of assistance they sought within the CPS program, this analysis examined sociodemographic characteristics by case type. As a benefits investigation case was typically initiated for new patients entering the program (98.5%; n=4,496), the characteristics of this group mirrored those of the overall group. Patients who sought appeals support after coverage denial (15.0%; n=687) were evenly distributed among age groups. As in the overall group, many patients lived in the South (48.8%; 335/687); however, the majority of patients who sought appeals support were from other geographic regions. In particular, 27.7% (190/687) of appeals support patients were from the Midwest. This group also had the highest percentage of Medicare beneficiaries (47.7%; 328/687).

Patients enrolled in the Free Medication Program were predominantly younger; 78.2% (1051/1344) of this group was below 65 years of age. Additionally, these patients were more likely to have no insurance than the overall group (52.8% [710/1344] vs. 16.6% [755/4566], respectively) or to be Medicaid beneficiaries (11.8% [158/1344] vs. 8.7% [397/4566], respectively).

Patients requesting Commercial Co-pay Program assistance were typically female (77.5%; 286/369) and young (aged <65: 93.8%; 346/369); most received healthcare in community settings (94.9%; 350/369). As this program was developed for commercially insured patients, it is not surprising that these patients were predominantly younger compared to the overall group.

Compared to other case types, the proportion of patients whose providers requested assistance with prior authorization or precertification was extremely low (1.3%; n=58). Most of these patients were commercially insured (58.6%; 34/58), young (aged <65 years: 63.8%; 37/58), female (69.0%; 40/58), and seen in a community setting (86.2%; 50/58).

**Service Utilization**

The frequency of service use by case type for patients enrolled in the CPS program to gain access to nab-paclitaxel as prescribed by their providers is presented in Table 2 and Table 3. Since benefits investigations were typically conducted for all patients entering the CPS program, these were the most common case type; other case types, which would follow those benefits investigations, were less common. Benefits investigations were 59.5% of all CPS cases (4836/8134); the Free Medication Program was 21.6% (1757/8134); appeals support 13.2% (1077/8134); the Commercial Co-pay Program 5.0% (405/8,134); and support to obtain prior authorization/precertification <0.8% (59/8134).

**Table 1.** Sociodemographic Characteristics of Patients by Case Type[1]

| | | Case Type[2] | | | | |
|---|---|---|---|---|---|---|
| **Characteristics** | **All** | **Benefits Investigation** | **Prior Authorization** | **Patient Assistance Program** | **Appeals Support** | **Commercial Co-Pay Program Assistance** |
| **Number of Patients** | 4566 | 4496 (98.5%) | 58 (1.3%) | 1344 (29.4%) | 687 (15.0%) | 369 (8.1%) |
| **Number of Cases per Patient** | | | | | | |
| Mean (SD) | 1.8 (1.2) | 1.8 (1.2) | 3.0 (2.5) | 2.8 (1.4) | 3.1 (1.8) | 2.5 (1.1) |
| **Age** | | | | | | |
| Mean (SD) | 61.7 (11.9) | 61.7 (11.9) | 60.8 (12.0) | 57.6 (11.5) | 63.7 (11.5) | 55.7 (8.6) |
| **Age Group, No. (%)** | | | | | | |
| <65 years | 2704 (59.2%) | 2670 (59.4%) | 37 (63.8%) | 1051 (78.2%) | 353 (51.4%) | 346 (93.8%) |
| 65+ years | 1862 (40.8%) | 1826 (40.6%) | 21 (36.2%) | 293 (21.8%) | 334 (48.6%) | 23 (6.2%) |
| **Gender, No. (%)** | | | | | | |
| Female | 2953 (64.7%) | 2912 (64.8%) | 40 (69.0%) | 915 (68.1%) | 426 (62.0%) | 286 (77.5%) |
| Male | 1606 (35.2%) | 1580 (35.1%) | 18 (31.0%) | 427 (31.8%) | 258 (37.6%) | 83 (22.5%) |
| Unspecified | 7 (0.2%) | 4 (0.09%) | 0 (0.0%) | 2 (0.1%) | 3 (0.4%) | 0 (0.0%) |
| **Geographic Region, No. (%)** | | | | | | |
| West | 709 (15.5%) | 702 (15.6%) | 9 (15.5%) | 243 (18.1%) | 103 (15.0%) | 90 (24.4%) |
| Midwest | 872 (19.1%) | 844 (18.8%) | 11 (19.0%) | 222 (16.5%) | 190 (27.7%) | 66 (17.9%) |
| South | 2459 (53.9%) | 2429 (54.0%) | 33 (56.9%) | 706 (52.5%) | 335 (48.8%) | 180 (48.8%) |
| Northeast | 516 (11.3%) | 511 (11.4%) | 5 (8.6%) | 168 (12.5%) | 59 (8.6%) | 33 (8.9%) |
| Missing | 10 (0.2%) | 10 (0.2%) | 0 (0.0%) | 5 (0.4%) | 0 (0.0%) | 0 (0.0%) |
| **Provider Type, No. (%)** | | | | | | |
| Academic | 554 (12.1%) | 541 (12.0%) | 8 (13.8%) | 227 (16.9%) | 105 (15.3%) | 19 (5.1%) |
| Community | 4012 (87.9%) | 3955 (88.0%) | 50 (86.2%) | 1117 (83.1%) | 582 (84.7%) | 350 (94.9%) |

HMO: health maintenance organization; PPO: preferred provider organization; SD: standard deviation

[1]Table provides patient-level information with frequencies referring to the number of patients.

[2]Columns are not mutually exclusive since patients may have more than one case.

**Table 1.** Sociodemographic Characteristics of Patients by Case Type[1]

| | | Case Type[2] | | | | |
|---|---|---|---|---|---|---|
| **Characteristics** | **All** | **Benefits Investigation** | **Prior Authorization** | **Patient Assistance Program** | **Appeals Support** | **Commercial Co-Pay Program Assistance** |
| **Number of Patients** | 4566 | 4496 (98.5%) | 58 (1.3%) | 1344 (29.4%) | 687 (15.0%) | 369 (8.1%) |
| **Number of Cases per Patient** | | | | | | |
| Mean (SD) | 1.8 (1.2) | 1.8 (1.2) | 3.0 (2.5) | 2.8 (1.4) | 3.1 (1.8) | 2.5 (1.1) |
| **Age** | | | | | | |
| Mean (SD) | 61.7 (11.9) | 61.7 (11.9) | 60.8 (12.0) | 57.6 (11.5) | 63.7 (11.5) | 55.7 (8.6) |
| **Age Group, No. (%)** | | | | | | |
| <65 years | 2704 (59.2%) | 2670 (59.4%) | 37 (63.8%) | 1051 (78.2%) | 353 (51.4%) | 346 (93.8%) |
| 65+ years | 1862 (40.8%) | 1826 (40.6%) | 21 (36.2%) | 293 (21.8%) | 334 (48.6%) | 23 (6.2%) |
| **Gender, No. (%)** | | | | | | |
| Female | 2953 (64.7%) | 2912 (64.8%) | 40 (69.0%) | 915 (68.1%) | 426 (62.0%) | 286 (77.5%) |
| Male | 1606 (35.2%) | 1580 (35.1%) | 18 (31.0%) | 427 (31.8%) | 258 (37.6%) | 83 (22.5%) |
| Unspecified | 7 (0.2%) | 4 (0.09%) | 0 (0.0%) | 2 (0.1%) | 3 (0.4%) | 0 (0.0%) |
| **Geographic Region, No. (%)** | | | | | | |
| West | 709 (15.5%) | 702 (15.6%) | 9 (15.5%) | 243 (18.1%) | 103 (15.0%) | 90 (24.4%) |
| Midwest | 872 (19.1%) | 844 (18.8%) | 11 (19.0%) | 222 (16.5%) | 190 (27.7%) | 66 (17.9%) |
| South | 2459 (53.9%) | 2429 (54.0%) | 33 (56.9%) | 706 (52.5%) | 335 (48.8%) | 180 (48.8%) |
| Northeast | 516 (11.3%) | 511 (11.4%) | 5 (8.6%) | 168 (12.5%) | 59 (8.6%) | 33 (8.9%) |
| Missing | 10 (0.2%) | 10 (0.2%) | 0 (0.0%) | 5 (0.4%) | 0 (0.0%) | 0 (0.0%) |
| **Provider Type, No. (%)** | | | | | | |
| Academic | 554 (12.1%) | 541 (12.0%) | 8 (13.8%) | 227 (16.9%) | 105 (15.3%) | 19 (5.1%) |
| Community | 4012 (87.9%) | 3955 (88.0%) | 50 (86.2%) | 1117 (83.1%) | 582 (84.7%) | 350 (94.9%) |

HMO: health maintenance organization; PPO: preferred provider organization; SD: standard deviation

[1]Table provides patient-level information with frequencies referring to the number of patients.

[2]Columns are not mutually exclusive since patients may have more than one case.

**Table 2.** Case Outcomes by Insurance Type[1]

| | Insurance Type | | | | | | | | |
|---|---|---|---|---|---|---|---|---|---|
| | **All** | **Medicare** | **Medicaid** | **Commercial w/HMO/PPO** | **Military (Tricare)** | **State Program** | **No Insurance** | **Other Commercial** | **Other/ Missing** |
| **Number of Cases** | 8134 | 2640 | 629 | 2,850 | 99 | 29 | 1777 | 0 | 114 |
| **Benefits Investigation (BI)** | | | | | | | | | |
| Assistance No Longer Needed | 109 (1.3%) | 51 (1.9%) | 8 (1.3%) | 39 (1.4%) | 2 (2.0%) | 0 (0.0%) | 9 (0.5%) | 0 (0.0%) | 0 (0.0%) |
| Assistance Provided | 806 (9.9%) | 298 (11.3%) | 73 (11.6%) | 289 (10.1%) | 14 (14.1%) | 5 (17.2%) | 110 (6.2%) | 0 (0.0%) | 17 (14.9%) |
| Benefits Available | 2529 (31.1%) | 1116 (42.3%) | 171 (27.2%) | 1125 (39.5%) | 49 (49.5%) | 8 (27.6%) | 16 (0.9%) | 0 (0.0%) | 44 (38.6%) |
| Benefits Not Available | 1392 (17.1%) | 267 (10.1%) | 108 (17.2%) | 319 (11.2%) | 4 (4.0%) | 5 (17.2%) | 686 (38.7%) | 0 (0.0%) | 3 (2.6%) |
| **Patient Assistance Program (PAP)** | | | | | | | | | |
| Free Drug: Prospective PAP (before treatment) | | | | | | | | | |
| Favorable | 1142 (14.0%) | 178 (6.7%) | 129 (20.5%) | 104 (3.6%) | 1 (1.0%) | 7 (24.1%) | 721 (40.6%) | 0 (0.0%) | 2 (1.7%) |
| Unfavorable | 144 (1.8%) | 23 (0.9%) | 10 (1.6%) | 21 (0.7%) | 0 (0.0%) | 2 (6.9%) | 87 (4.9%) | 0 (0.0%) | 1 (0.9%) |
| Free Drug: Replacement PAP (after treatment) | | | | | | | | | |
| Favorable | 428 (5.3%) | 123 (4.7%) | 47 (7.5%) | 121 (4.2%) | 2 (2.0%) | 1 (3.4%) | 129 (7.3%) | 0 (0.0%) | 5 (4.3%) |
| Unfavorable | 43 (0.5%) | 16 (0.6%) | 2 (0.3%) | 13 (0.5%) | 0 (0.0%) | 0 (0.0%) | 12 (0.7%) | 0 (0.0%) | 0 (0.0%) |
| **Prior Authorization/Precertification (PA)** | | | | | | | | | |
| Authorization Granted | 31 (0.4%) | 13 (0.5%) | 2 (0.3%) | 16 (0.6%) | 0 (0.0%) | 0 (0.0%) | 0 (0.0%) | 0 (0.0%) | 0 (0.0%) |
| Authorization Denied | 11 (0.1%) | 4 (0.2%) | 0 (0.0%) | 6 (0.2%) | 1 (1.0%) | 0 (0.0%) | 0 (0.0%) | 0 (0.0%) | 0 (0.0%) |

BI: benefits investigation; HMO: health maintenance organization; PA: prior authorization/precertification; PAP: patient assistance program; PPO: preferred provider organization

[1]Table provides case level information with frequencies referring to the number of cases.

**Table 3.** Case Outcomes by Case Type[1]

| Case Outcomes | Case Types |
|---|---|
| Total Number of Cases | 8134 |
| **Appeals Support[2]** | |
| **Case Type** | **Appeals Support[2]** |
| Number of Cases | 1077 |
| Total Reimbursed (across all appeal levels) | 455 |
| Total Not Reimbursed (across all appeal levels) | 265 |
| 1st Level Appeal, No. (%) | |
| Reimbursed | 275 (25.5%) |
| Not Reimbursed | 144 (13.4%) |
| Reason for 1st Level Appeal Failure | |
| Additional Information Needed | 1 (0.09%) |
| Exceeds Dosage Restrictions | 3 (0.3%) |
| No Coverage Under Policy | 3 (0.3%) |
| Not Medically Necessary | 120 (11.1%) |
| Unspecified | 17 (1.6%) |
| 2nd Level Appeal, No. (%) | |
| Reimbursed | 173 (16.1%) |
| Not Reimbursed | 117 (10.9%) |
| Reason for 2nd Level Appeal Failure | |
| No Coverage Under Policy | 1 (0.09%) |
| Not Medically Necessary | 97 (9.0%) |
| Not In Combo With Specified TX | 1 (0.09%) |
| Unspecified | 18 (1.7%) |
| 3rd Level Appeal, No. (%) | |
| Reimbursed | 7 (0.6%) |
| Not Reimbursed | 4 (0.4%) |
| Reason for 3rd Level Appeal Failure | |
| Not Medically Necessary | 4 (0.4%) |
| **Patient Assistance Program (PAP)** | |
| **Case Type** | **Patient Assistance Program** |
| Number of Cases | 1757 |
| Free Drug: Prospective PAP (before treatment) | |
| Favorable | 1142 (65.0%) |
| Unfavorable | 144 (8.2%) |
| Free Drug: Replacement PAP (after treatment) | |
| Favorable | 428 (24.4%) |
| Unfavorable | 43 (2.4%) |

**Table 3.** Case Outcomes by Case Type[1] (continued)

| Case Outcomes | Case Types |
|---|---|
| **Co-payment Assistance** | |
| **Case type** | **Co-payment Assistance** |
| Number of Cases | 405 |
| Co-pay Cases with Commercial Insurance | |
| Grant from Commercial Program: Favorable | 378 (93.3%) |
| No.(%) Case Reporting | 231 (57.0%) |
| Mean(SD) Grant, $ | 597 (639) |
| Median (Interquartile Range) Grant, $ | 412 (169–746) |
| Grant from Commercial Program: Unfavorable | 27 (6.7%) |
| **Prior Authorization/Precertification (PA)** | |
| **Case Type** | **Prior Authorization/ Precertification (PA)** |
| Number of Cases | 59 |
| Authorization Granted | 31 (52.5%) |
| Authorization Denied | 11 (18.6%) |
| Reason Authorization Granted | |
| Unspecified | 31 (52.5%) |
| Reason Authorization Denied | |
| No Coverage Under Policy | 2 (3.4%) |
| Not Medically Necessary | 8 (13.6%) |
| Prior Regimen Not Tried/Failed | 1 (1.7%) |
| **Benefits Investigation (BI)** | |
| **Case Type** | **Benefits Investigation (BI)** |
| Number of Cases | 4836 |
| Assistance No Longer Needed | 109 (2.3%) |
| Assistance Provided | 806 (16.7%) |
| Benefits Available | 2529 (52.3%) |
| Benefits Not Available | 1392 (28.8%) |

BI: benefits investigation; PA: prior authorization/precertification; PAP: patient assistance program; TX: prescription

[1]Table provides case level information with frequencies referring to the number of cases.

[2] Outcomes for appeals support cases that are not tabulated include: assistance no longer needed (n=328), assistance provided (n=28), and remand denial (n=1). Due to the exclusion of selected case outcomes, the sum of the appeals does not equal the total number of cases.

#### Effect of the Patient Support Program

More than half or 52.3% (2,529/4,836) of benefits investigation cases determined that patients had existing insurance benefits available to them (Table 3). Among the cases where no patient benefits were available, 49.3% (686/1,392) of cases had no insurance, 19.2% (267/1,392) had Medicare, 22.9% (319/1,392) had commercial insurance, and 7.8% (108/1,392) had Medicaid (Table 2).

The CPS program effectively managed support for cases of drug reimbursement appeals after coverage denial. Of 1,077 appeals support cases initiated, 720 were resolved after first-, second-, or third-level appeals; in almost all of the remaining cases (328), appeals support was eventually no longer needed (Table 3). Of the 720 cases resolved with CPS program support, payer reimbursement for nab-paclitaxel was obtained in 455 (63.2%) of the cases; 38.2% (275/720) of cases were reimbursed on the first appeal, 24.0% (173/720) on second appeal, and 1.0% (7/720) on third appeal (Table 3).

Based on a financial assessment, CPS staff evaluated patients for their possible eligibility for these programs. Most patients meeting financial criteria[16] and initially thought likely to be eligible for these programs received assistance (Free Medicine Program: 89.4% of cases, 1570/1757; Commercial Co-pay Program: 93.3% of cases; 378/405; Table 3). Fewer cases of patients with commercial insurance coverage received free medicine (12.8%; 225/1757) than those with either Medicare (17.1%; 301/1757) or no insurance (48.4%; 850/1757; Table 2), suggesting that commercial insurance is somewhat more likely to provide coverage for nab-paclitaxel than is Medicare. Among patients who received copayment support from the Commercial Co-pay Program, the mean (SD) amount of support granted was $597 (639) per case (Table 3).

As previously mentioned, support with prior authorization was the least frequently used service within the CPS program. While few healthcare providers requested assistance with prior authorization (1.3%; 58/4566), the majority of prior authorizations assisted by CPS were approved (53.4%; 31/59) (Table 3). Of the 11 cases denied authorization, most were among patients with commercial insurance (n=6; Table 2). The remainder of the cases denied authorization were among patients on Medicare (n=4) and one patient on Military (Tricare) insurance (Table 2).

### DISCUSSION

The present study documented the need for and the positive effect of a patient support program supporting patient access to treatment by providing assistance maneuvering through the healthcare reimbursement system. As demonstrated by this analysis of patients being treated for solid tumor malignancies, a diverse group of patients may opt into a manufacturer-sponsored program with comprehensive service offerings. CPS specialists helped patients explore all options available to ensure better overall health outcomes. The CPS program assisted patients across a representative spectrum of insurance types and coverage status. Most participating patients were insured by Medicare (38.5%) or commercial plans (37.3%), while fewer patients had no insurance (16.6%) or were Medicaid beneficiaries (8.7%). The study shows that many patients need assistance even to access coverage that they already have, which suggests that insurers could improve communications with their enrolled beneficiaries. The fact that many patients also end up needing financial assistance, through either co-pay assistance or free medicine, suggests that there is a need for more comprehensive insurance coverage among patients in the US.

This evaluation demonstrated that benefits investigations can be effectively implemented by a patient support program. The CPS benefits investigation service provides valuable assistance in navigating the complex healthcare insurance environment to providers and to patients who may not be aware of the details of their insurance coverage or their eligibility for various support programs. During the almost three year study, the CPS program determined that patients had some form of insurance coverage in more than half (52.3%) of benefit investigation cases. In such cases, the CPS program was able to offer assistance with prior authorization or precertification, support for appeals, and consideration for the Commercial Co-Pay Program for those meeting financial criteria. When the CPS program could not identify available benefits, it was most often in cases where patients had no insurance, although there were some patients with Medicare, Medicaid, or commercial insurance plans who did not have available benefits.

Assistance obtaining free medicine continues to be a frequently requested and successful support service for cancer patients in the United States. Approximately 22% of CPS program cases were requests for help accessing free medicine, and most (89.4%) of the cases that met all the financial criteria did, in fact, receive free medicine.

Few cases managed by the CPS program were for appeals support, commercial co-pay program assistance, or prior authorization. Support with appeals for drug reimbursement after a coverage denial was effectively managed by the CPS program, with 63.2% of cases resolved receiving reimbursement for nab-paclitaxel and with over a third reimbursed on the first appeal. With the success of the appeals support service, the CPS program may effectively ease the burden of healthcare providers whose offices previously had devoted substantial time and effort to patients who sought drug reimbursement after coverage denial.[3]

While the Commercial Co-pay Program is less frequently used, 93.3% of the cases that CPS opened for patients who met the financial criteria resulted in financial support for co-pays and out-of pocket expenses. The success rate suggests this offering is a valuable solution for patient access problems. For commercial co-pay cases where the outcome was unfavorable, the CPS program was able to offer other services, such as referrals to third-party copay and transportation assistance programs to help defray out-of-pocket costs.

This study contributes to the limited body of evidence indicating that patient support programs successfully improve access to care for cancer patients. A previous analysis of CPS program offerings for patients with hematologic malignancies demonstrated that comparable proportions of such patients enrolled in the support program as those who were not enrolled received their prescribed oral cancer therapy.[3] While the effectiveness of commercial co-pay program assistance for US cancer patients was demonstrated in a previous analysis, that study did not examine a program with service offerings beyond financial support, nor a patient population with diverse sociodemographics and insurance types.[4]

The present study cohort does represent the heterogeneity of US cancer patients, who exhibit diverse sociodemographic and socioeconomic status, provider types, and health insurance coverage. Additionally, this study describes how patients use support services available to them, sometimes more than one service, and how these support services are effective in providing access to care. Despite its effectiveness, the CPS program is limited by a general lack of awareness and utilization. A Celgene report shows that only 19% of accounts, a mix of community and academic-based practices that use nab-paclitaxel, used the CPS program in 2013. An increase in CPS program utilization will likely yield more successful patient outcomes.

There are several limitations of the analyses that should be considered when interpreting patient characteristics, service use, and outcomes data. First, the research design was retrospective and observational. Future assessments of these types of data should be conducted to see if this pattern of results continues, or perhaps changes as different aspects of federal health policy change. Individual US states are implementing portions of the Patient Protection and Affordable Care Act[19,20] differently from one another; this provides the potential for "natural experiments" to be conducted, comparing patients and assistance results under different rules in different states. Future assessment of the need for and outcomes of these patient support services would be valuable, to both observe trends and confirm whether positive outcomes persist. Second, there was no control group of patients who were prescribed nab-paclitaxel but not enrolled in the CPS program. Without a direct comparison to cancer patients who lacked access to support services, the true impact of the CPS program relative to other means of obtaining these types of assistance is difficult to quantify. That said, since few organizations offer patient support similar to the CPS program, the availability of these offerings could be an effective component of any broader set of such patient support services. Also, as the study only provides information on patients enrolled in the CPS program, no information could be obtained on patients who did not seek support through CPS program and could not access nab-paclitaxel therapy out of those who were prescribed. Third, the data for the cases receiving financial support in obtaining medicine may not represent all patients who sought such support as this study's available data was based on patients who met all financial criteria. Future assessments should attempt to quantify the reasons for lack of eligibility, and their prevalence.

### CONCLUSION

The findings suggest that the CPS program was successful overall by effectively providing assistance through various service components. The CPS program was valuable for US cancer patients in gaining access to therapy prescribed by their healthcare providers. Given the complexity of US health insurance and continual rise of patient out-of-pocket costs, such a program will be a valuable resource for helping patients and their healthcare providers understand health insurance benefits and access other services such as appeals support, co-pay assistance, and/or free medicine. Although there was a general lack of utilization of the CPS program, increase in awareness of such a patient support program will help patients achieve overall successful outcomes.

### ACKNOWLEDGEMENTS

The authors acknowledge Juliet Brines Hommes, Jillian Fahey, and Julie Graybill of Celgene Corporation for providing access to data and making this study possible. Kathy Tater, Yingxin Xu, Talia Foster, and Janet Dooley are acknowledged for their assistance in manuscript preparation. Celgene Patient Support program staff members are acknowledged for their services on behalf of the patients whose experience has been documented here.

#### Conflict of Interest Declaration

Celgene Corporation of Summit, New Jersey, USA funded the study and this resulting manuscript. Zeba M. Khan is an employee and stockholder of Celgene Corporation. Alissa Shaul, and Brian Murphy are current employees of Evidera, and Lindsay Parker was an employee of Evidera at the time of the study conduct and manuscript creation. Evidera provides consulting and other research services to pharmaceutical, device, government, and non-government organizations. In their salaried positions, Evidera employees work with a variety of companies and organizations and are precluded from receiving payment or honoraria directly from these organizations for services rendered. Evidera received funding from Celgene Corporation for the conduct of this study and the writing of this manuscript.
